# A Genomic and Proteomic Approach to Identify and Quantify the Expressed *Bacillus thuringiensis* Proteins in the Supernatant and Parasporal Crystal

**DOI:** 10.3390/toxins10050193

**Published:** 2018-05-10

**Authors:** Joaquín Gomis-Cebolla, Ana Paula Scaramal Ricietto, Juan Ferré

**Affiliations:** 1 ERI de Biotecnología y Biomedicina (BIOTECMED), Department of Genetics, Universitat de València, 46100 Burjassot, Spain; Joaquin.Gomis@uv.es (J.G.-C.); ricietto@gmail.com (A.P.S.R.); 2 Departamento de Biologia Geral, Universidade Estadual de Londrina, Londrina 86057-970, Paraná, Brazil

**Keywords:** insect pest control, crop protection, vip proteins, cry proteins

## Abstract

The combined analysis of genomic and proteomic data allowed us to determine which *cry* and *vip* genes are present in a *Bacillus thuringiensis* (*Bt*) isolate and which ones are being expressed. Nine *Bt* isolates were selected from Spanish collections of *Bt* based on their *vip1* and *vip2* gene content. As a first step, nine isolates were analyzed by PCR to select those *Bt* isolates that contained genes with the lowest similarity to already described *vip1* and *vip2* genes (isolates E-SE10.2 and O-V84.2). Two selected isolates were subjected to a combined genomic and proteomic analysis. The results showed that the *Bt* isolate E-SE10.2 codifies for two new vegetative proteins, Vip2Ac-like_1 and Sip1Aa-like_1, that do not show expression differences at 24 h vs. 48 h and are expressed in a low amount. The *Bt* isolate O-V84.2 codifies for three new vegetative proteins, Vip4Aa-like_1, Vip4Aa-like_2, and Vip2Ac-like_2, that are marginally expressed. The Vip4Aa-like_1 protein was two-fold more abundant at 24 h vs. 48 h, while the Vip4Aa-like_2 was detected only at 24 h. For Vip2Ac-like_2, no differences in expression were found at 24 h vs. 48 h. Moreover, the parasporal crystal of the E-SE10.2 isolate contains a single type of crystal protein, Cry23Aa-like, while the parasporal crystal from O-V84.2 contains three kinds of crystal proteins: 7.0–9.8% weight of Cry45Aa-like proteins, 35–37% weight of Cry32-like proteins and 2.8–4.3% weight of Cry73-like protein.

## 1. Introduction

*Bacillus thuringiensis* (*Bt*) is an entomopathogenic bacterium that produces several types of insecticidal proteins, such as Cry, Cyt, Vip, Sip, Mtx-like, and Bin-like proteins, along with other virulence factors contributing to its pathogenicity [1,2]. The Vip proteins are a family of proteins that are secreted during the vegetative growth phase and that have been classified into four groups according to their sequence homology: Vip1, Vip2, Vip3, and Vip4 [2]. Because of repeated applications of *Bt* sprays and the widespread adoption of *Bt*-crops (transgenic crops protected from insects by the expression of *cry* and/or *vip3* genes), some insect populations have developed resistance to *Bt* toxins [3,4,5,6]. Therefore, in this arms race against insects, it is necessary to explore the potential of new insecticidal proteins for pest control. A series of approaches have been used for isolating novel insecticidal protein genes from *Bt*, such as PCR, which has further evolved into specific applications to mining new insecticidal genes, such as PCR hybridization, PCR-RFLP, E-PCR and PCRSSCP [7,8,9,10,11]. In addition, the construction of *Bt* DNA libraries, followed by screening by Western Blotting or a hybridization-based method, has also been used to detect novel insecticidal protein genes [12,13,14]. The PCR approaches being used to detect *vip* genes are based on the presence of conserved blocks in the DNA sequence of these genes [11,15] and most of the studies have focused on genes from the *vip3* family. Therefore, the PCR approach is limited to finding *vip* genes with enough homology to the primers used. An additional problem with the PCR approach is that it does not provide the full length of the new *vip* genes. On the other hand, the library-based methods are time-consuming and laborious. The next generation sequencing (NGS) allows rapid sequencing of entire genomes at a low cost-effective ratio [16,17]. The number of *Bt* whole genomes that have been sequenced has increased quickly in the past decade. To date, 459 *Bt* strains have been sequenced, with a mean genome size ranging from 5.3 MB to 6.7 MB and a mean guanine-cytosine content (GC content) between 34% and 35% (https://www.ncbi.nlm.nih.gov/genome/?term=Bacillus+thuringiensis). The combination of the low cost NGS with the development of many freeware tools has enabled the rapid detection of insecticidal protein genes at the genome level. On the other hand, the development of the mass spectrometry (MS)-based proteomics has enabled the detection of proteins from complex mixtures from different stages of a microorganism [18]. The combination of the genomic and proteomic approaches is a very successful approach for validation and correction of predicted genomic coding information in a wide variety of organisms [18,19,20,21,22].

In this study, the identity of *vip* genes has been determined in nine *Bt* isolates which were candidates to harboring new *vip1* and *vip2* genes (Vip1 and Vip2 constitute a binary toxin and their genes are normally located in an operon). Two of these isolates, which were found to carry *vip*-type genes with a similarity lower than 45% to already reported genes, were subjected to whole genome sequencing and to different kinds of proteomic analysis to determine and estimate the relative abundance of the expressed insecticidal protein genes.

## 2. Results

### 2.1. Identification of Vip1-, Vip2-, and Vip4-Type Genes

To identify the specific genes within the *vip1* and *vip2* gene families, a strategy based on PCR-Sanger Sequencing was used. A first PCR with “screening primers” was performed to confirm the presence of *vip* genes. The results showed that the nine isolates were positive for the presence of a *vip1-type* gene, and that seven were positive for the presence of a *vip2-type* gene (Table 1). Those samples that gave positive for a determined gene type were subjected to a second PCR with “typing primers” to narrow down the identity of the gene. The results allowed us to classify the isolates into two types of isolates containing a *vip1–vip2* gene pair: those with a gene pair with high similarity (>95%) to *vip2Bb* (KR065728)–*vip1Bb* (KR065727) (V-J20.2 and V-LE1.1), and those with a gene pair with high similarity to *vip2Ac* (KR065726)–*vip1Ca* (KR065725) (V-V54.26, V-V54.31, E-TE7.43, E-TE16.5 and E-TE18.40). In addition to these two categories, two isolates were identified to contain just a single *vip* gene with low similarity to all reported ones; one had the highest similarity to *vip1Bb* (E-SE10.2) and the other had the highest similarity to *vip1Da* (O-V84.2), which was later shown to belong to the *vip4Aa* family (Table 1).

### 2.2. Genome Sequencing of the Bt Isolates E-SE10.2 and O-V84.2, Contig Assembly and Gene Annotation

Whole genome sequencing of the *Bt* isolates E-SE10.2 and O-V84.2 resulted in 10,401,436 high quality reads for the *Bt* isolate E-SE10.2 and 9,210,116 high quality reads for the *Bt* isolate O-V84.2, with an average length of 150 bp for both *Bt* isolates. For the E-SE10.2 isolate, the 97.4% of the reads were assembled in 222 scaffolds while for the O-V84.2 isolate the 98.2% of the reads were assembled in 249 scaffolds. For the E-SE10.2 isolate, the results of the assembled paired reads were as follows: genome size of 6.1 Mb, N50 was 71 kb, the GC content 36%, and the longest scaffold length was 258 kb. For the O-V84.2 isolate, the genome size was 6.3 Mb, N50 was 123 kb, the GC content 36%, and the longest scaffold length was 336 kb. Coding sequence prediction of the assembled reads showed that the 222 scaffolds of the E-SE10.2 isolate defined 6156 coding sequences (CDS) and that the 249 scaffolds of the O-V84.2 isolate defined 6457 CDS. For both isolates, the CDS represented the 79% of the length of the bacterial genome, and contained 71.5% of annotated genes, 28.5% of hypothetical genes, and 60–68 tRNAs (Table 2). In addition, for both isolates, 60% of the CDS could be associated to a subsystem category, being more abundant the ones associated with amino acids and derivatives, carbohydrates, protein metabolism, and cofactors, prostetic groups and pigments, in decreasing order (Figure S1).

Regarding the insecticidal protein genes present in the *Bt* isolates E-SE10.2 and O-V84.2, the results indicated a total of 24 coding sequences (6 in E-SE10.2 and 18 in O-V84.2) (Table 3). For the *Bt* isolate E-SE10.2, four out of the six sequences showed homology to a *vip* gene, one to a *sip* gene and one to a *cry* gene (Table 3). In the case of the *Bt* isolate O-V84.2, 10 out of the 18 sequences showed homology to a *vip* gene, one to a *sip* gene and seven to a *cry* gene (Table 3).

### 2.3. Global Analysis of the Proteins Identified by in Gel Digestion LC/MSMS Analysis of the Bt Isolates E-SE10.2 and O-V84.2

To determine the proteins that are being expressed in the *Bt* isolates E-SE10.2 and O-V84.2, an LC/MSMS analysis was done. By this method, we first screened the protein content in the concentrated supernatants and in the parasporal crystals at three growth phases, two during the log phase of growth (Phase T1 at 24 h and Phase T2 at 48 h), and one in the stationary phase when the crystal is formed (Phase T3 at 72 h). In the concentrated supernatant at Phase T1, 627 and 225 proteins were identified for E-SE10.2 and O-V84.2, respectively, while, at Phase T2, the number of proteins identified were 637 and 530, respectively. In the case of the proteins identified in the solubilized crystal (Phase T3), the numbers were 512 and 185, respectively. A total of 1791 and 940, respectively, were identified considering the three growth phases together and this represents about the 29.03% and 14.55% of the respective predicted proteins from the genomic data for the *Bt* isolates E-SE10.2 and O-V84.2. The pairwise comparison of the identified proteins at the T1, T2 and T3 growth phases showed that the shared expressed proteins of the T1-T2, T2-T3, and T1-T3 phases were 406 and 160, 221 and 73, 219 and 33, respectively, for the *Bt* isolates E-SE10.2 and O-V84.2. The identified proteins of *Bt* isolates E-SE10.2 and O-V84.2 at the three growth phases were classified according to their gene ontology (GO) terms (Figure S2).

### 2.4. Protein Identification of the Expressed Predicted Putative Insecticidal Protein Genes

To determine if the predicted insecticidal protein genes are being expressed, the protein expression was assessed by proteomic analysis. We considered a positive identification only those proteins that were identified with both Protein Pilot v4.5 and Mascot algorithms in at least two of the replicates. Considering the two isolates together, we found a total of five secretable proteins (Vip-like and Sip-like) and eight crystal proteins (Cry-like and Mtx-like) (Table 4 and Tables S1–S3). For the E-SE10.2 isolate, only three out of the six putative insecticidal protein genes automatically annotated were found to be expressed (Table 3), and 10 out of 18, in the case of the O-V84.2 isolate (the seven Cry proteins, the Vip2Ac-like_2 protein, and the two Vip4-like proteins). Regarding the Vip2Ac-like_3 protein, it was detected just in one replicate with Mascot (Table 4 and Table S3) and the Sip1Aa-like_2 protein was detected in two replicates, but in one of them only with Mascot (Table 4, Tables S1 and S3) and, therefore, the Vip2Ac-like_3 and Sip1Aa-like_2 proteins were not considered a positive identification.

According to the similarity to the closest homolog, the Vip2Ac-like, Vip4Aa-like, Cry32Aa-like and Sip1Aa-like_2 proteins could be considered new *Bt*-like proteins (different to Cry, Vip or Sip because of a similarity lower than 45%). Regarding the Cry45Aa-like, Cry73Aa-like, Cry23Aa-like and Sip1Aa-like-1 proteins, according to the similarity to the closest homolog (between 45% and 75%), they could be considered new protein families of their respective reference proteins (e.g., with a different number) (Table 3). Regarding the subcellular localization of the putative insecticidal proteins, we performed an LC/MSMS analysis with the concentrated culture supernatants and with the solubilized crystal proteins (Table 4). In the supernatant of the culture broth, we could detect at 24 h and 48 h the Vip2Ac-like_1, Sip1Aa-like_1 and Cry23Aa-like proteins in the *Bt* isolate E-SE10.2. For the *Bt* isolate O-V84.2, we detected Vip4Aa-like_1, Vip4Aa-like_2 and Vip2Ac-like_2 proteins at 24 h, whereas, at 48 h, we detected Vip4Aa-like_1 and Vip2Ac-like_2 proteins (Table 4). In the fraction of solubilized crystal proteins, we detected the Cry23Aa-like protein in the *Bt* isolate E-SE10.2, while, for the *Bt* isolate O-V84.2, we detected the Cry45Aa-like_1, Cry45Aa-like_2, Cry45Aa-like_3, Cry32Ea-like, Cry32Eb-like, Cry32Da-like and Cry73Aa-like proteins. The putative insecticidal proteins identified in the supernatant and solubilized crystal agree with the prediction of the SignalIP server 4.0, except for the Cry23Aa-like protein which has been found in the supernatant and in the crystal even though it does not contain signal peptide to be exported out of the cell (Table 4).

### 2.5. Gene synteny, Conserved Domains and Phylogenetic Analysis of the Expressed Putative Insecticidal Protein Genes

**Figure 1:**
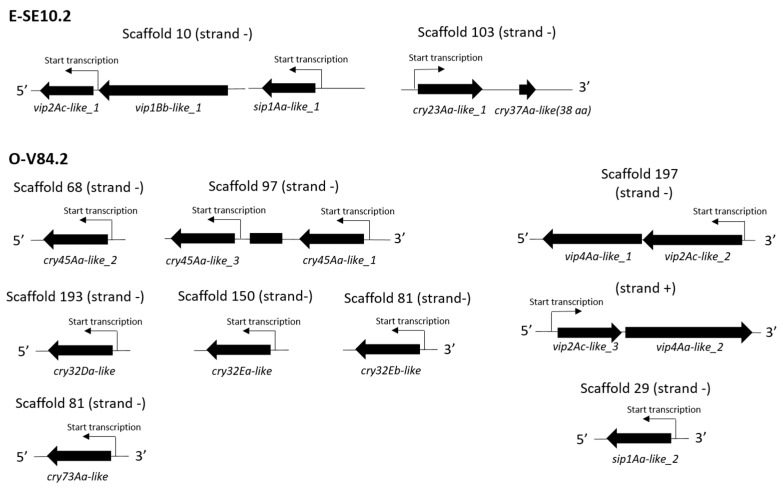
Gene synteny of the putative insecticidal protein genes of the E-SE10.2 and O-V84.2 isolates detected by LC/MSMS analysis. Strand + indicate that the respective genes are in the positive DNA strand. Strand—means that the respective genes are in the negative DNA strand.

In the E-SE10.2 isolate, the *vip2Ac-like_1* gene was found in an operon together with a non-expressed *vip1Bb-like* gene, with the peculiarity that the *vip1Bb-like* gene was upstream of the *vip2Ac-like_1* (Figure 1), contrary to the general relative location of *vip1* and *vip2* genes in operons. The *cry32Aa-like* gene was found in an operon with a predicted truncated *cry37-like* gene. In the O-V84.2 isolate, the genes for the Vip2Ac-like_2, Vip4Aa-like_1, and Vip4Aa-like_2 proteins were found in operons containing the pairs *vip2Ac-like_2*–*vip4Aa-like_1* and *vip2Ac-like_3*–*vip4Aa-like_2* (Figure 1). Regarding the *cry* genes of this isolate, they were found in different scaffolds with different transcription origins (Figure 1).

**Figure 2:**
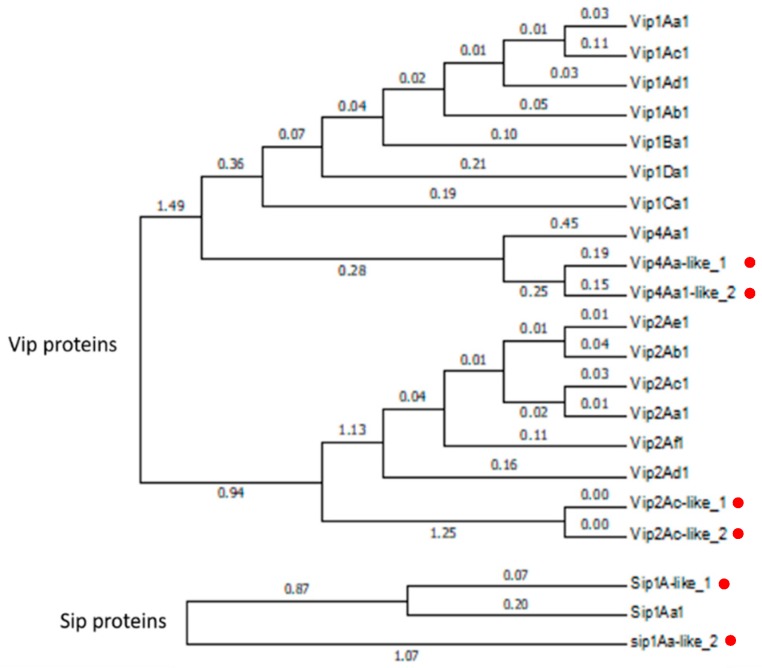
Phylogenetic analysis of the Vip1/Vip2- and Sip1A-type proteins detected in the *Bt* isolates E-SE10.2 and O-V84.2. The red dots indicate the position of the new putative proteins in the phylogenetic tree. Branch lengths represent the number of substitutions per site of the multiple-sequence alignment as a measure of divergence (Mega v6 software).

**Figure 3:**
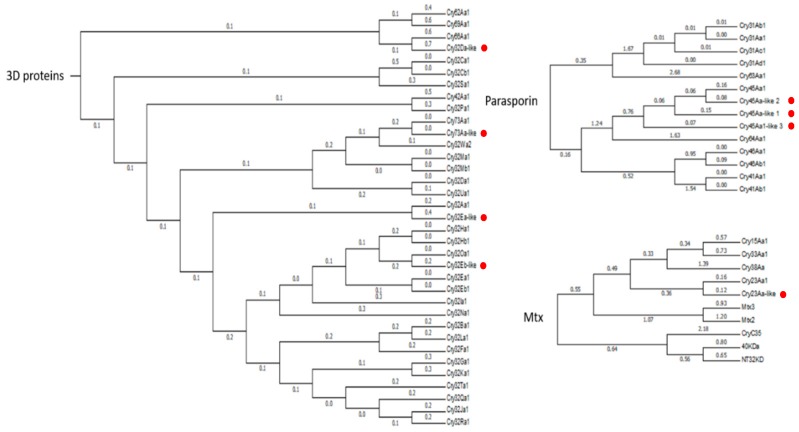
Phylogenetic analysis of the Cry-type proteins detected in the *Bt* isolates E-SE10.2 and O-V84.2. The red dots indicate the position of the new putative proteins in the phylogenetic tree. Branch lengths represent the number of substitutions per site of the multiple-sequence alignment as a measure of divergence (Mega v6 software).

The phylogenetic analysis of the Vip-like and Sip1-like proteins indicate that the Vip4-like, Vip2-like, and Sip1Aa-like_2 create a basal branch in their respective families (Figure 2). Regarding the Cry proteins, the Cry32E-like, Cry45Aa-like, Cry73Aa-like, and Cry23Aa-like proteins fall into their respective clusters, whereas the Cry32Da-like protein falls into the Cry66A cluster (Figure 3). The analysis of the sequences revealed that the *vip4Aa-like_1* and *vip4Aa-like_2* showed the predicted conserved domains of the PA14 superfamily and the clostridial binary toxin B/anthrax toxin PA, which are present in the Vip1 proteins. The *vip2Ac-like_1*, *vip2Ac-like_2* and *vip2Ac-like_3* genes showed the predicted conserved domain Vip2 superfamily, which is present in the Vip2 proteins. Moreover, *vip2Ac-like_2* showed the predicted conserved domain anthrax toxin lethal factor (ATLF) and *vip2Ac-like_3* showed one of the two Vip2 superfamily conserved domains present in the Vip2 proteins. The *sip1Aa-like* genes (*sip1Aa-like_1* and *sip1Aa-like_2*) showed the predicted conserved domain of the MTX superfamily which is present in the *Lysinibacillus sphearicus* and *Clostridium perfringens*. Regarding the Cry-like proteins, the analysis of the sequences revealed that the *cry23Aa*-*like* gene and the *cry45Aa-like* genes (*cry45Aa-like_1*, *cry45Aa-like_2*, and *cry45Aa-like_3* have a similarity of 75% to each other) showed the predicted conserved domain MTX. Among the *cry32-like* genes, *cry32Ea-like* and *cry32Da-like* showed a similarity of 88% to each other (at the amino acid level) and both carried the predicted conserved domains Endotoxin_N, Endotoxin_M, and Delta_Endotoxin_C, which are typical of the three-domain Cry proteins. The *cry32Eb-like* gene showed low similarity to the other two *cry32-like* genes (61% amino acid similarity to *cry32Ea-like* and 64% amino acid similarity to *cry32Da-like*) and did not show any conserved domains. The *cry73Aa-like* gene also showed the predicted conserved domains Endotoxin_N, Endotoxin_M, and Delta_Endotoxin_C.

### 2.6. Relative Abundance of the Putative Insecticidal Proteins in the Supernatant and in the Crystal of the Bt Isolates E-SE10.2 and O-V84.2

To determine the relative abundance of the putative insecticidal protein genes in the supernatants and crystals of the *Bt* isolates E-SE10.2 and O-V84.2, we performed two types of analyses: first, an emPAI analysis to determine the relative abundance within a same replicate at a given time (Table 5 and Table S3); and, second, a label free analysis to compare between different times in the log phase (T1 vs. T2) (Table 6 and Table S4). The results showed that the putative vegetative insecticidal proteins were minimally expressed in the supernatant of both *Bt* isolates, being the most abundant protein flagellin FlaA (Table 5). In the *Bt* isolate E-SE10.2, among the putative insecticidal proteins found in the supernatant, the most abundant in all replicates was the Cry23Aa-like protein. In contrast, for O-V84.2, all secretable proteins were similarly represented (Table 5). Regarding the relative abundance of the proteins in the solubilized crystals, the crystal of E-SE10.2 contained only the Cry23Aa-like protein. In the case of O-V84.2, the percent weight corresponding to Cry proteins was close to the 50% of the solubilized proteins from the crystal (Table 5), being the most abundant, by far, the Cry32Ea-like protein.

To be able to compare the expression level of the proteins between 24 h and 48 h, a label free analysis was performed (Table 6 and Table S4). Only the putative insecticidal proteins Vip4Aa-like_1 and Vip4Aa-like_2, from the *Bt* isolate O-V84.2, showed significant differences at the two growth phases (Table 5 and Table 6). The former increased two-fold at 48 h compared to 24 h (Table 6), and the latter was only found at 24 h but not at 48 h (Table 5). The other proteins found in the supernatant did not show statistical differences in their production at 24 h vs. 48 h.

## 3. Discussion

A screening of Spanish collections of *Bt* isolates was undertaken to search for novel members of the Vip family. As a result, nine *Bt* isolates were selected for harboring new binary insecticidal protein genes of the *vip1/vip2* family [11]. As a first step, the PCR-Sanger Sequencing approach revealed new alleles of already described *vip1* and *vip2* genes (*vip2Ac2-vip1Ca2* and *vip2Bb4-vip1Bb3*) and two sequences with low similarity to the *vip1Bb1* (from the *Bt* isolate E-SE10.2) and *vip4Aa1* (from the *Bt* isolate O-V84.2) genes. In a second step, the *Bt* isolates E-SE10.2 and O-V84.2 were subjected to whole genome sequencing with the Illumina HiSeq-PE150 sequencing platform. Then, the genomes of E-SE10.2 and O-V84.2 were assembled in 222 and 249 scaffolds codifying for 6156 CDS and 6457 CDS, respectively. The CDS predicted for both genomes represented close to the 99% of the total number of genes predicted in the genomes. In addition, from this 99% of the predicted CDS, 28% belonged to hypothetical genes and 72% to annotated genes by the Rast server. Moreover, the results obtained from the automated annotation indicated that both *Bt* genomes had a similar subsystems category distribution (Figure S1).

The supernatants at 24 h (growth Phase T1) and 48 h (growth Phase T2) and the crystal proteins (growth Phase T3) of both *Bt* isolates were also analyzed and annotated with GO terms (Figure S2). The quantity of the proteins expressed at the three different growth phases for the *Bt* isolates E-SE10.2 and O-V84.2 were 10.2–3.5% and 10.4–8.2%, 8.3–2.8%, respectively, of their genome encoded sequences. This low percentage of expressed proteins detected indicates that, in our experimental conditions, we only detect a small part of the predicted proteins by the genome data prediction, a phenomenon that has also been found in other studies [21,22]. The low percentage of detected expressed proteins should not be interpreted as that the rest of the proteins cannot be expressed, since they could do it under different growth conditions. Considering both isolates together, the number of annotated proteins in each growth phase, T1, T2 and T3, was 42.3%, 49.8% and 56.9%, respectively. The distribution of the GO terms over the different growth phases is similar in the *Bt* isolates E-SE10.2 and O-V84.2 (Figure S2). The most common and abundant GO terms in all the phases (cellular biosynthetic process, organic substance biosynthetic process, cellular nitrogen compound metabolic process, and organonitrogen compound metabolic process) indicate that both *Bt* isolates metabolize the carbon and nitrogen in the media to produce all the organic and organonitrogen compounds that they need (Figure S2). The specific GO term macromolecule metabolic process of the T3 growth phase indicates that both *Bt* isolates express proteins of a relatively high molecular mass, such as the Cry-like proteins detected (Figure S2).

Regarding the predicted insecticidal protein genes in both *Bt* genomes, we were able to find some of the predicted gene products: one new couple of binary Vip-like proteins (Vip2Ac-like_1-Vip4Aa-like_1), two new Vip-like proteins (Vip2Ac-like_1 and Vip4Aa-like_2), one Sip1A-like protein (Sip1A-like_1), and eight Crystal-like proteins (Cry23A-like, Cry45Aa-like_1, Cry45Aa-like_2, Cry45Aa-like_3, Cry32Ea-like, Cry32eDa-like, Cry32Eb-like and Cry73Aa-like) (Table 4). The discrepancies of the protein identification between the replicates can be attributed to metabolic flow changes in cells during development, resulting from enzyme-related changes or that some proteins exist with extremely low abundances such that they cannot be detected by MS. To determine if the detected *Bt-like* proteins are being secreted or that they form inclusion bodies, we performed an LC/MSMS analysis with the supernatant (24 h and 48 h) and solubilized crystal proteins. In the supernatant of both *Bt* isolates at 24 h, the Vip4Aa-like_1, Vip4Aa-like_2, Vip2Ac-like_1, Vip2Ac-like_2, and Sip1Aa-like_1 proteins were detected, while at 48 h only Vip4Aa-like_1, Vip2Ac-like_1, Vip2Ac-like_2, and Sip1Aa-like_1 were detected. Again, the extremely low abundance of these proteins might be responsible for the differences found at 24 h and 48 h. Regarding the Vip4Aa-like proteins, this is the first time that there has been demonstrated that they are expressed and secreted to the medium in the log phase. Regarding the crystal proteins, they were found in the crystal of both *Bt* isolates, except for the Cry23Aa-like, which was also found in the supernatant at 24 h and 48 h. The detection of the Cry23Aa-like protein and sporulation factors (Stage V sporulation protein, spore coat protein B, spore coat polysaccharide biosynthesis protein spsB and spore coat polysaccharide synthesis) in the supernatant at 24 h (and also at 48 h) of the *Bt* isolate E-SE10.2 indicates that the cells already started the sporulation process.

The relative abundance of the *Bt-like* proteins was estimated in the supernatant and the parasporal crystal in both *Bt* isolates. In the supernatant (24 h and 48 h) of both *Bt* isolates, the Vip-like, Sip1-like and Cry23Aa-like were marginally expressed. Regarding the crystal proteins in the *Bt* isolate O-V84.2, the Cry-like proteins represent around the half of the total crystal weight, while for the *Bt* isolate E-SE10.2, the Cry23Aa-like protein represents between 2.5% and 30% of the crystal weight. The high variability observed in the amount of Cry23Aa-like could be due to the different replicates are not in the same time point of the sporulation process. The crystal composition of the *Bt* isolate O-V84.2 was also determined for those proteins with a percentage of similarity lower than 45%. The crystal was composed by four kinds of proteins: 7.0–9.8% Cry45-like proteins (Cry45Aa-like_1, Cry45Aa-like_2 and Cry45Aa-like_3), 30.4–30.5% Cry32-like proteins (Cry32Ea-like and Cry32Da-like), 5.0–6.2% Cry32Eb-like, and 2.8–4.25% Cry73Aa-like, while the *Bt* isolate E-SE10.2 only produced the Cry23Aa-like protein.

The expression levels of the Vip-like, Sip1-like and Cry23Aa-like proteins were compared between 24 h and 48 h. The amount of Vip4Aa-like_1 protein was increased two-fold at 24 h vs. 48 h, while the Vip4Aa-like_2 was only detected at 24 h. As regard to the rest of the proteins (Vip2Ac-like, Sip1A-like, and Cry23A-like proteins), no differences in expression were observed. These results suggest that the Vip4Aa-like_2, Vip2A-like and Sip1A-like proteins were expressed at the 24 h while the Vip4Aa-like_1 was expressed later at the end of the 24 h and the beginning of the 48 h periods.

## 4. Conclusions

In summary, the combined use of the genomic and proteomic data allowed us to determine which of the identified insecticidal protein genes, present in the *Bt* isolates E-SE10.2 and O-V84.2, are being expressed and, if so, at which relative abundance. Considering the two *Bt* isolates together, we were able to identify five new insecticidal protein genes that are expressed within the first 24 h, except for *vip4Aa-like*_*1*, which is expressed after the 24 h. In the parasporal crystals, we found nine new crystal proteins. The spore/crystal mixture of the *Bt* isolate E-SE10.2 contains solely the Cry23Aa-like protein, while the crystal of the *Bt* isolate O-V84.2 contains four kinds of Cry proteins: Cry45-like, Cry32-like, Cry32Eb-like, and Cry73Aa-like.

## 5. Materials and Methods

### 5.1. Bacterial Strains and Growth Conditions for DNA Analysis

Nine *Bt* isolates from a Spanish collection, known to carry *vip1* and *vip2* genes, were selected for this study [11]. For further gene identification of the *vip1* and *vip2* genes, the *Bt* isolates were grown in 4 mL LB medium overnight (ON) at 29 °C and 200 rpm. For the whole genome sequencing, only those *Bt* isolates with *vip1* and *vip2* genes with less than 60% similarity to already described *vip1* and *vip2* genes were chosen. The isolates were grown in 10 mL LB medium until OD of 0.6 at 29 °C and 200 rpm.

### 5.2. Genomic DNA Preparation

Total genomic DNA used for gene identification (GI) was isolated from a single colony of the *Bt* isolates. Cells were collected at 9000× *g* for 10 min at 4 °C and the pellet was washed in 2 mL of TE buffer (1 M Tris-HCl, 10 mM EDTA, pH 8.0). The pellet was dissolved in 200 μL of TEL buffer (TE buffer + 4 mg/mL lysozyme) and further incubated at 37 °C for 30 min. Then, 400 μL of lysis solution (0.2 M NaOH, 1% SDS) was added. After gentle mixing, 300 μL of the neutralization buffer (3 M KAc, pH 5.5) was added and the mixture incubated for 5 min on ice. The mixture was centrifuged at 14,000× *g* for 15 min at 4 °C and the supernatant was transferred to a new tube. One volume of cold 100% ethanol was added and the samples kept at −20 °C for 16 h. The samples were centrifuged at 14,000× *g* for 15 min at 4 °C and the supernatant was transferred to a new tube and the pellet washed with 1 mL of cold 70% ethanol. The pellet was dried with the Eppendorf concentrator 5301 for 5 min at 42 °C and solubilized in 50 μL of TE buffer. Total genomic DNA, used for whole genome sequencing (WGS), was purified as described in the manufacturer instructions of the DNeasy Blood & Tissue Kit Qiagen. The DNA for GI was quantified using Nanodrop 2000 (Thermo Scientific, Waltham, MA, USA), while for WGS, the DNA was measured with a Qubit Fluorimetrer. In addition, the integrity of the DNA for GI and WGS was evaluated by agarose gel electrophoresis (1% agarose).

### 5.3. Identification of Vip1- and Vip2-Type Genes

Identification of *vip1* and *vip2* genes was performed with primer pairs designed from conserved regions within the *vip1* and *vip2* gene families, respectively. A first PCR with “screening primers” [11] was performed to confirm the presence of *vip* genes. With the positive samples, a second PCR was performed with the “typing primers” [11,23] for the identification of the *vip1* and *vip2* genes. PCR reactions contained, in a final volume of 25 μL, 100 ng of the DNA template, 0.25 U of Biotools polymerase (Biotools), 2.5 μL of 10-fold reaction buffer, 10 mM of each dNTPs, and 0.3 µM of the corresponding primers (*vip1sc*, *vip2sc*, *vip2 typing* [11] or *vip1 typing* [23]). PCR amplifications were carried out in an Eppendorf Mastercycler thermal cycler as follows: 5 min denaturation at 95 °C, 35 cycles of amplification (1 min denaturation at 94 °C, 1 min of annealing at 45 °C, and 2 min of extension at 72 °C), and an extra extension step of 10 min at 72 °C. To determine the similarity of the amplified sequences to already described *vip1* and *vip2* genes, the PCR products obtained with the “typing primers” (or with the “screening primers” for those samples that did not give amplification with the “typing primers”) were ligated into the pGEM^®^-T Easy plasmid (Promega), cloned in *Escherichia coli* DH10β, and sequenced. DNA sequence analysis and contig assembly was performed using DNAstar v5 and NCBI BLAST tools (Blastx) [24].

### 5.4. Genome Sequencing, Assembly and Annotation Analysis

Genome Sequencing for the *Bt* isolates E-SE10.2 and O-V84.2 was performed with the Illumina HiSeq-PE150 sequencing platform (Novogene S.L Hong Kong, China). From the clean reads (without adapters, low quality, N and duplication) provided by Novogene S.L., first we evaluated the quality of the data with FastQC software (0.11.5, Babraham Bioinformatics Institute, Cambridge, Cambridgeshire, United Kingdom, 2016). Then, the reads were assembled with SoapdeNovo2 (kmer size 35 and genome size 5,600,000 bp) and the gaps were closed with GapCloser (maximum read length 150, overlap 25 bp and thread number 1) [25]. The assembled reads were annotated with Rast server (Figure S1) and the coding sequence (CDS) prediction was performed with the Glimmer v2 [26]. First, the predicted genes were filtered against a customized *Bt* protein database (https://sourceforge.net/projects/bt-proteindatabase/files/Btdatabase/) with Blastx (genetic code bacteria and archaea, e-value 0.001 and word size 6) to select those CDS with homology to the *Bt* toxins [24]. Next, the putative insecticidal protein genes were compared against the Non-Redundant database and only the concordant results along the customized *Bt* protein database and Non-Redundant database were selected as true positive. Moreover, for the selected putative insecticidal genes, prediction of conserved domains was carried out with CD-search [27] and the gene sinteny was determined in the assembled sequences.

### 5.5. Sample Preparation for in Gel Digestion LC/MSMS Analysis and Insecticidal Activity of Bt Isolates

A single colony of *Bt* was grown in 100 mL of LB at 29 °C for 24 h and 48 h for detection of the secretable proteins, while for the detection of proteins in the parasporal crystal the culture was grown in 100 mL of CCY at 29 °C until culture sporulation (72 h). The supernatant of *Bt* was concentrated by trichloroacetic acid (TCA) precipitation. Briefly, the cells were collected at 6000× *g* for 15 min at 4 °C and filtered through sterile 0.45 μm cellulose acetate filters (GE Healthcare Life Sciences). The sample was incubated with 10% TCA (final concentration) and kept at 4 °C for 24 h. Then, the sample was centrifuged at 16,000× *g* for 20 min at 4 °C. The pellet was washed with 100 mL of cold acetone (−18 °C), centrifuged at 16,000× *g* for 20 min at 4 °C, and let dry at room temperature for 5 min. The precipitated proteins were solubilized in 50 mM carbonate buffer containing 10 mM dithiothreitol (pH 11.3) for 48 h, with two buffer changes (Figure S3). Crystals (together with spores) were separated by centrifugation at 6000× *g* for 12 min at 4 °C. The pellet containing the parasporal crystals was washed three times with ice cold solution A (1 M NaCl, 5 mM EDTA, 10 mM PMSF, 1% Triton X-100) and centrifuged at 17,000× *g* for 12 min at 4 °C between washes. The pellet was then washed three times with ice cold solution B (10 mM KCl) and centrifuged at 24,000× *g* for 15 min at 4 °C. The crystals in the final pellet were solubilized in 20 mL of 50 mM carbonate buffer containing 10 mM dithiothreitol (pH 11.3) by incubation at room temperature for 2 h with continuous shaking (Figure S3). Concentration of the proteins in the supernatant and in the solubilized crystals was estimated with the Bradford method [28]. The purity of the expressed proteins in the supernatant and the crystal was analyzed by SDS-PAGE and stained with Coomassie brilliant blue R-250 (Sigma-Aldrich, St. Louis, MO, USA) (Figure S3).

### 5.6. In Gel Digestion LC/MSMS Analysis

The detection of the expression of the putative insecticidal proteins was done by LC/MSMS at the proteomics facility of the SCSIE (Servei Central de Suport a la Investigació Experimental), at the University of Valencia, Spain. First, a 1D SDS-PAGE (without resolving gel) was performed with 30 µg of total protein in three replicates of the concentrated supernatant (24 h and 48 h) and solubilized crystal proteins. The bands were cut out and in gel digested with 500 ng sequencing grade trypsin (Promega). The digestion was stopped with trifluoroacetic acid (TFA, 1% final concentration). After subjecting the samples to a double extraction with acetonitrile (ACN), all the peptide solutions were dried in a rotatory evaporator. Samples were solubilized with 50 μL of 2% ACN, 0.1% TFA. A sample aliquot of 5 μL was loaded onto a trap column (NanoLC Column, 3 µ C18-CL, 350 µm × 0.5 mm, Eksigent) and desalted with 0.1% TFA at 3 μL/min for 5 min. The peptides were then loaded onto an analytical column (LC Column, 3 µ C18-CL, 75 µm × 12 cm, Nikkyo) equilibrated in 5% ACN 0.1% formic acid (FA). The elution was carried out with a linear gradient of 5–35% B in A for 30 min (A: 0.1% FA; B: ACN, 0.1% FA) at a flow rate of 0.3 μL/min. Peptides were analyzed in a nanoESI qQTOF (5600 TripleTOF, ABSCIEX) mass spectrometer. Eluted peptides were ionized applying 2.8 kV to the spray emitter. Analysis was carried out in a data-dependent mode (DDA). Survey MS1 scans were acquired from 350 to 1250 *m*/*z* for 250 ms. The quadrupole resolution was set to “UNIT” for MS2 experiments, which were acquired 100–1500 *m*/*z* for 50 ms in “high sensitivity” mode. The following switch criteria were used: charge: 2+ to 5+; minimum intensity; 70 counts per second (cps). Up to 25 ions were selected for fragmentation after each survey scan and the collision energy was automatically selected by the instrument according to the following equation: |CE| = (slope) × (*m*/*z*) + (intercept); Charge (Unknown, 1, 2, 3, 4, 5), Slope (0.0575, 0.0575, 0.0625, 0.0625, 0.0625, 0.0625), Intercept (9, 9, −3, −3, −6, −6)

### 5.7. Protein Identification of the in Gel Digestion LC/MSMS Analysis with Paragon Algorithm and Mascot

The MS/MS information of three replicates of the concentrated supernatant (24 h and 48 h) and solubilized crystal proteins were sent to Paragon algorithm [29] via the Protein Pilot v 4.5 (ABSciex). Protein Pilot v 4.5 default parameters were used to generate peak list directly from the 5600 TripleTof Sciex. The Paragon algorithm of Protein Pilot v 4.5 was used to search in a homemade database that was created combining all the coding sequences predicted by Glimmer v2 software for the *Bt* isolates E-SE10.2 and O-V84.2; the new database was named *Bt*_combined (https://sourceforge.net/projects/bt-combined/files/Bt_combined/). The search in the respective protein database was done with the following parameters: trypsin specificity, cys-alkylation, and the search effort set to through. To avoid using the same spectral evidence in more than one protein, the identified proteins were grouped based on MS/MS spectra (proteins sharing MS/MS spectra are grouped, regardless of the peptide sequence assigned) by the Protein-Pilot Progroup algorithm. A protein group in a Progroup Report is a set of proteins that share some physical evidence, the formation of protein groups in Pro Group was guided entirely by observed peptides only and the unobserved regions of protein sequence play no role in explaining the data (Tables S1 and S2). The protein within each group which can explain more spectral data is that protein shown as the primary protein of the group. Only the proteins of the group for which there is individual evidence (unique peptides with enough confidence) are also listed (Tables S1 and S2). In addition, to support the identification of the Protein Pilot v 4.5 (ABSciex) and estimate the relative production of the insecticidal proteins in the three replicates of the concentrated supernatant and solubilized crystal proteins, a series of Mascot MS/MS ion searches with the output of the 5600 TripleTof Sciex were done with the *Bt*_combined protein database. The following parameters were used: MS/MS “ion search”, enzyme “trypsin”, fixed modifications “carbamidomethyl (C)”, variable modifications “deamidated (NQ) and oxidation (M)”, mass values “monoisotopic”, protein mass “unrestricted”, peptide mass tolerance “50 ppm”, fragment mass tolerance “0.6 Da”, max miss cleavages “1”, instrument type “ESI-QUAD-TOF”, number of queries for E-SE10.2 “(Supernatant 24 h: R1 7468, R2 8755, R3 7682; Supernatant 48 h: R1 9, 243, R2 8602, R38,286; Crystal: R1 6779, R2 7173 R3 7790)” and for O-V84.2 “(Supernatant 24 h: R1 3708, R2 4459, R3 3536 Supernatant 48 h: R1 6016, R2 6654, R3 6123; Crystal: R1 5206 R2 5206 R3 4476)”, significance threshold “*p*-value < 0,05”, max number of families “auto”, ions score or expect cut-off “20”, and preferred taxonomy “all entries”. The Exponentially Modified Protein Abundance Index (emPAI) was expressed as molar and weight percentage [30] (Table 5 and Table S3).

We defined as a true positive all those proteins with homology to the *Bt* toxins higher than 100 aa that had been identified with Protein Pilot v4.5 and Mascot in at least two of the replicates. In addition, for the identified proteins, the functional annotation was performed with the SwissProt Database using the Blast2GO v5.0 software (Figure S2) [31].

### 5.8. Label Free Analysis of the Concentrated Supernatant 24 h vs. 48 h in Both Bt Isolates

The data obtained from the 5600 TripleTof Sciex of the concentrated supernatant and solubilized proteins from the crystal were analyzed by Peak View 1.1 following the parameters: Unused ≥ 1.3, confidence > 95% and with maximum 50 peptides for protein. For the protein library construction of the global analysis, a joint search with the *Bt*_combined protein database was performed with the three replicates of the concentrated supernatant (24 h and 48 h) and solubilized crystal proteins (Table S5). In the case of the specific conditions analysis (Supernatant: E-SE10.2 24 h vs. 48 h, and O-V84.2 24 h vs. 48 h), a joint search with the *Bt*_combined protein database was performed with the three replicates of the concentrated supernatant (Table S4). The search in the respective analysis was done with the following parameters: trypsin specificity, cys-alkylation, and the search effort set to through. First, a global analysis was done to study grouped data analysis and samples distribution. A joined search with all the samples was performed with the Peak View 1.1 that identified 1816 proteins and the quantitative data obtained was analyzed with Marker View 1.3. Briefly, for the grouped data analysis, a PCA analysis was done with the non-normalized area of the peaks and with the area peaks corrected by the total areas sum. In the case of the samples distribution, a PCA analysis was done with the area of the peaks corrected by the total areas sum (Table S5). For the specific conditions analysis, a specific search with Peak View 1.1 was done with the respective samples to study the statistical significant differences. The quantitative data was analyzed with Marker View 1.3. Prior to data analysis of the E-SE10.2 24 h vs. 48 h, and O-V84.2 24 h vs. 48 h, we applied a normalization by total areas sum, and then a grouped data analysis with PCA analysis was done. A student’s *t*-test statistical analysis with the concentrated supernatant (E-SE10.2 24 h vs. 48 h, and O-V84.2 24 h vs. 48 h) was performed to determine the differentially expressed proteins between two experimental conditions with the Marker View 3.1 software (Table S4).

## Figures and Tables

**Table 1:** Identification of *vip1*, *vip2* and *vip4* genes in selected isolates of *Bacillus thuringiensis.*

Name of Isolate	Identified with *vip1* Primers			Identified with *vip2* Primers		
	Similarity (%) ^‡^	Coverage (%) *	Closest Homolog	Similarity (%) ^‡^	Coverage (%) *	Closest Homolog
V-J20.2	100	44	*vip1Bb1*	97	70	*vip2Bb1*
V-LE1.1	100	40	*vip1Bb1*	99	72	*vip2Bb1*
V-V54.26	99	49	*vip1Ca1*	99	71	*vip2Ac1*
V-V54.31	100	49	*vip1Ca1*	98	73	*vip2Ac1*
E-SE10.2	62	30	*vip1Bb3*	No DNA amplification		
E-TE7.43	100	49	*vip1Ca1*	99	64	*vip2Ac1*
E-TE16.5	98	43	*vip1Ca1*	98	73	*vip2Ac1*
E-TE18.40	100	30	*vip1Ca1*	100	45	*vip2Ac1*
O-V84.2	40	40	*vip4Aa1*	No DNA amplification		

* The coverage values represent the mean of two replicates of the typing or screening PCR products to the full sequence length of the respective *vip1*, *vip2* and *vip4* genes deposited in GenBank. ^‡^ The similarity values provided by BlastX (NCBI) represent the mean value of the in silico translation of two replicates of the PCR products.

**Table 2:** Summary of the automated genome annotation of the *Bt* isolates E-SE10.2 and O-V84.2 by the Rast server.

Features	E-SE10.2		O-V84.2	
	Gene Content	Length (Mb)	Gene Content	Length (Mb)
Genome Content *	6216	6.1	6525	6.3
Coding sequences ^‡^	6156 (99%)	4.8	6457 (98.9%)	5
Annotated genes	4398 (70%)	4.05	4615 (71.4%)	4.21
Hypothetical genes	1758 (28.2%)	0.75	1842 (28.2%)	0.79
Predicted insecticidal genes ^§^	6	0.002	18	0.4
tRNAs	60	0.004	68	0.005

* The gene content values refer the total of predicted sequences (Coding sequences and tRNAs) predicted by the Rast server. ^‡^ The coding sequences values refer to the total predicted sequences (protein encoding genes and rRNA). The annotated genes refer to the predicted sequences that were included in subsystem category, while the hypothetical genes refer to the predicted sequences that were not included in any subsystem category. The percentage of coding sequences was calculated by dividing the values of the coding sequences, annotated genes and hypothetical genes, by the value of genome content. ^§^ The predicted insecticidal genes refer to the coding sequences that report similarity to the homemade *Bt* database at amino acid level (BlastX).

**Table 3:** Insecticidal genes of the E-SE10.2 and O-V84.2 isolates predicted by Glimmer v2 software and filtered against a customized *Bt* protein database and then with the non-redundant database (NCBI).

Sample	Gene Identity ^‡^	Closest Homolog *	Similarity (%)	Coverage (%)
E-SE10.2	*vip1Ad-like_1*	AGC08395.1	55	24
	*vip1Bb-like_1*	AAR40282.1	61	99
	*vip2Aa-like*	1QS1_A	41	23
	*vip2Ac-like_1*	AAO86513.1	30	30
	*sip1Aa-like_1*	ABC71340.1	75	98
	*cry23Aa-like*	AAF76375.1	75	98
O-V84.2	*vip1Ad-like_2*	AGC08395.1	26	34
	*vip1Ba-like*	AAR40886.1	28	30
	*vip1Da-like*	CAI40767.1	37	12
	*vip2Ac-like_2*	AAO86513.1	33	47
	*vip2Ac-like_3*	AAO86513.1	37	41
	*vip2Bb-like*	AKI69695.1	30	43
	*vip4Aa1-like_1*	AEB52299.1	40	80
	*vip4Aa1-like_2*	AEB52299.1	40	83
	*vip4Aa-like_3*	AEB52299.1	49	94
	*vip4Aa-like_4*	AEB52299.1	52	97
	*sip1Aa-like_3*	ABC71340.1	32	33
	*cry45Aa-like_1*	BAD22577.1	61	100
	*cry45Aa-like_2*	BAD22577.1	69	99
	*cry45Aa-like_3*	BAD22577.1	68	85
	*cry32Ea-like*	ADK66923.1	47	98
	*cry32Eb-like*	AGU13828.1	51	41
	*cry32Da-like*	BAB78603.1	40	98
	*cry73Aa-like*	AEH76822.1	88	80

^‡^ The genes predicted by the gene prediction software were named based on the homologous gene in the database that showed more identity and coverage in the BlastX. * Access number of the gene that showed the highest identity in the protein database considered in the analysis.

**Table 4:** Identification of expressed proteins from the identified putative insecticidal protein genes in the concentrated supernatant and in the solubilized proteins from the spore/crystal mixture of the *Bt* isolates E-SE10.2 and O-V84.2 by in gel digestion LC/MSMS analysis *.

Sample	Protein Identity	Mass Protein (kDa)	SignalIP Server 4.1	Supernatant (LB)						Spore/Crystal Mixture (CCY)		
				24 h			48 h			72 h		
				Rep. 1	Rep. 2	Rep. 3	Rep. 1	Rep. 2	Rep. 3	Rep. 1	Rep. 2	Rep. 3
E-SE10.2	Vip2Ac-like_1	51.6	Yes	**+/+**	**+/+**	**+/+**	**+/+**	**+/+**	**+/+**	−/−	−/−	−/−
	Sip1Aa-like_1	40.7	Yes	**+/+**	**+/+**	**+/+**	**+/+**	**+/+**	**+/+**	−/−	−/−	−/−
	Cry23Aa-like	29.3	No	**+/+**	**+/+**	**+/+**	**+/+**	**+/+**	**+/+**	**+/+**	**+/+**	**+/+**
O-V84.2	Vip4Aa-like_1	97.5	Yes	**+/+**	**+/+**	**+/+**	**+/+**	**+/+**	**+/+**	−/−	−/−	−/−
	Vip2Ac-like_2	80.79	Yes	**+/+**	**+/+**	**+/+**	**−/+**	**+/+**	**+/+**	−/−	−/−	−/−
	Vip4Aa-like_2	87.5	Yes	**+/+**	**+/+**	**−/+**	−/−	−/−	−/−	−/−	−/−	−/−
	Vip2Ac-like_3	23.2	Yes	−/−	−/+	−/−	−/−	−/−	−/−	−/−	−/−	−/−
	Sip1Aa-like_2	38.7	Yes	−/−	−/+	+/+	−/−	−/−	−/−	−/−	−/−	−/−
	Cry45Aa-like_1	30.6	No	−/−	−/−	−/−	−/−	−/−	−/−	**+/+**	**+/+**	**+/+**
	Cry45Aa-like_2	29.3	No	−/−	−/−	−/−	−/−	−/−	−/−	**+/+**	**+/+**	**+/+**
	Cry45Aa-like_3	25.6	No	−/−	−/−	−/−	−/−	−/−	−/−	**+/+**	**+/+**	**+/+**
	Cry32Ea-like	151.2	No	−/−	−/−	−/−	−/−	−/−	−/−	**+/+**	**+/+**	**+/+**
	Cry32Da-like	153.7	No	−/−	−/−	−/−	−/−	−/−	−/−	**+/+**	**+/+**	**+/+**
	Cry32Eb-like	76.8	No	−/−	−/−	−/−	−/−	−/−	−/−	**+/+**	**+/+**	**+/+**
	Cry73Aa-like	72.2	No	−/−	−/−	−/−	−/−	−/−	−/−	**+/+**	**+/+**	**+/+**

* +/+, the insecticidal protein genes were identified with Protein Pilot (Paragon algorithm) and Mascot; +/−, the insecticidal protein genes were identified with Protein Pilot (Paragon algorithm) but not with Mascot; −/+, the insecticidal protein genes were identified with Mascot but not Protein Pilot (Paragon algorithm); −/−, the insecticidal protein genes were not identified with either Protein Pilot (Paragon algorithm) or Mascot.

**Table 5:** Estimation of the relative production expressed as weight percentage of the insecticidal protein genes in the supernatant and solubilized proteins from the spore and crystal mixtures of the *Bt* isolates E-SE10.2 and O-V84.2 identified with Mascot.

Supernatant	24 h (% Weight)			48 h (% Weight)		
	R1	R2	R3	R1	R2	R3
**E-SE10.2**						
*Non Secretable toxins*	98.56	99.06	99.36	97.87	99.26	99.63
Flagellin protein FlaA	55.21	73.82	87.19	39.75	36.04	43.19
*Secretable toxins*	1.44	0.94	0.64	2.13	0.74	0.37
Vip2Ac-like_1	0.09	0.06	0.03	0.09	0.10	0.06
Sip1A-like_1	0.09	0.03	0.02	0.06	0.08	0.02
Cry23Aa-like *	1.26	0.85	0.59	1.98	0.56	0.29
**O-V84.2**						
*Non Secretable toxins*	99.99	99.99	99.99	99.99	99.99	99.99
Flagellin protein FlaA	99.20	99.37	98.35	84.14	99.15	88.35
*Secretable toxins*	0.0032	0.0012	0.0030	0.0111	0.00007	0.0075
Vip4Aa-like_1	0.0014	0.0008	0.0016	0.0019	0.00004	0.0029
Vip4Aa-like_2	0.0005	0.0001	0.0002	-	-	-
Vip2Ac-like_2	0.0013	0.0002	0.0010	0.0092	0.00003	0.0046
**Crystal**	**72 h (% Weight)**					
	**R1**		**R2**		**R3**	
**E-SE10.2**						
*Non-crystal toxins*	69.52		95.26		97.51	
*Crystal toxins*	30.48		4.74		2.49	
Cry23A-like	30.48		4.74		2.49	
**O-V84.2**						
*Non-crystal toxins*	52.86		51.12		53.25	
*Crystal toxins*	47.14		48.88		46.75	
Cry45Aa-like_1	2.82		2.46		1.41	
Cry45Aa-like_2	3.05		2.03		3.38	
Cry45Aa-like_3	1.88		5.29		2.20	
Cry32Ea-like	24.30		25.06		25.89	
Cry32Da-like	6.10		5.40		4.60	
Cry32Eb-like	6.20		4.46		5.02	
Cry73Aa-like	2.79		4.18		4.25	

* The Cry23Aa-like protein was detected in the supernatant and the crystal of the *Bt* isolate E-SE10.2, but, according to the prediction of the SignalIP server 4.1, it is most likely not secretable.

**Table 6:** Label free analysis of the putative insecticidal protein genes of the *Bt* isolates E-SE102 and O-V84.2 in the concentrated supernatant at 24 h versus 48 h, identified with Protein Pilot v4.5.

*Bt* Isolate	Proteins	*t*-Value ^†^	*p*-Value ᶲ	Mean Peaks Area		Standard Deviation Peaks Area		Fold Change 24/48 ^§^	Status
				24 h	48 h	24 h	48 h		
E-SE10.2	Vip2Ac-like_1	0.71	0.52	307,838	266,602	54,005	84,577	1.15	No differences
	Sip1Aa-like_1	0.89	0.42	110,172	76,938	47,032	44,040	1.43	No differences
	Cry23Aa-like *	0.32	0.77	5,796,029	4,858,544	4,951,202	1,257,383	1.19	No differences
O-V84.2	Vip4Aa-like_1	4.07	0.04	134,357	68,825	26,801	7636	1.95	Increased
	Vip2Ac-like_2	0.56	0.61	32,544	27,573	13,419	7512	1.18	No differences

^†^ Student’s *t*-test statistical analysis was performed between the concentrated supernatant at 24 h versus 48 h. ᶲ With a p value lower than 0.05, it was considered that the differences observed between the concentrated supernatant at 24 h versus 48 h were statistically significant. ^§^ The fold change was calculated by dividing the mean value at 24 h by the mean values at 48 h. * The Cry23Aa-like protein was detected in the supernatant and the crystal of the *Bt* isolate E-SE10.2, but, according to the prediction of the SignalIP server 4.1, is most likely not secretable.

## Supplementary Materials

The following are available online at http://www.mdpi.com/2072-6651/10/5/193/s1, Figure S1: Subsystem category distribution of the *Bt* isolates E-SE10.2 and O-V84.2 by the genome annotation based on the Rast server. The right pie chart indicates the percentage or predicted encoding genes associated to at least one subsystem; Figure S2: Functional annotation of the protein identification from the concentrated supernatants at 24 h and 48 h in LB medium, and of the solubilized proteins from the crystal at 72 h in CCY medium. The number of sequences that belong to the group are indicated in brackets; Figure S3: SDS-PAGE of the three replicates of the concentrated supernatants and solubilized proteins from the spore/crystal mixtures and growth curve of the *Bt* isolates E-SE10.2 and O-V84.2; Table S1: Protein summary of three replicates of concentrated supernatants and solubilized proteins from spore/crystal mixtures with Protein Pilot v4.5; Table S2: Peptide summary of three replicates of concentrated supernatants and solubilized proteins from spore/crystal mixtures identified with Protein Pilot v4.5; Table S3: Result of the Mascot search in the three replicates of the concentrated supernatants and solubilized proteins form spore/crystal mixtures; Table S4: Label free analysis at 24 h vs. 48 h of concentrated supernatants of the proteins identified with Protein Pilot v4; Table S5: Protein library construction and PCA analysis of the joint search with the concentrated supernatants and solubilized proteins from spore/crystal mixtures identified with Protein Pilot v4.5.

## Availability of Data and Material

The clean reads of the *Bt* isolates have been deposited at Sequence Read Archive (SRA). E-SE10.2 SRR5115619 and O-V84.2 SRR5121303. The sequences of the genes have been deposited in the GenBank. V-J20.2: *vip1Bb3* (KR065727) and *vip2Bb4* (KR065728). V-V54.26: *vip1Ca2* (KR065725) and *vip2Ac2* (KR065726). E-SE10.2: vip2Ac-like_1 (KY420183), sip1Aa-like_1 (KY420184), cry23Aa-like (KY420185). O-V84.2: *vip4Aa-like_1* (KY420182), *vip4Aa-like_2* (KY420193), *vip2Ac-like_2* (KY420195), *vip2Ac-like_3* (KY420197), *cry45Aa-like_1* (KY420188), *cry45Aa-like_2* (KY420189), *cry32Ea-like* (KY420190), *cry32Da-like* (KY420191), *cry45Aa1-like_3* (KY420192), *cry73Aa-like* (KY420194), *cry32Eb-like* (KY420196).
