# Supplementary material for: A Genomic and Proteomic Approach to Identify and Quantify the Expressed Bacillus thuringiensis Proteins in the Supernatant and Parasporal Crystal
Source: Toxins (Basel). 2018 May 10;10(5):193. doi: 10.3390/toxins10050193 (PMC5983249; doi:10.3390/toxins10050193)
Supplement: Supplementary file 1 [file toxins-10-00193-s001.zip › toxins-298293 Supplementary.pdf]

Supplementary Materials: A Genomic and Proteomic Approach to Identify and Quantify the Expressed *Bacillus thuringiensis* Proteins in the Supernatant and Parasporal Crystal

Joaquín Gomis-Cebolla, Ana Paula Scaramal Ricietto and Juan Ferré

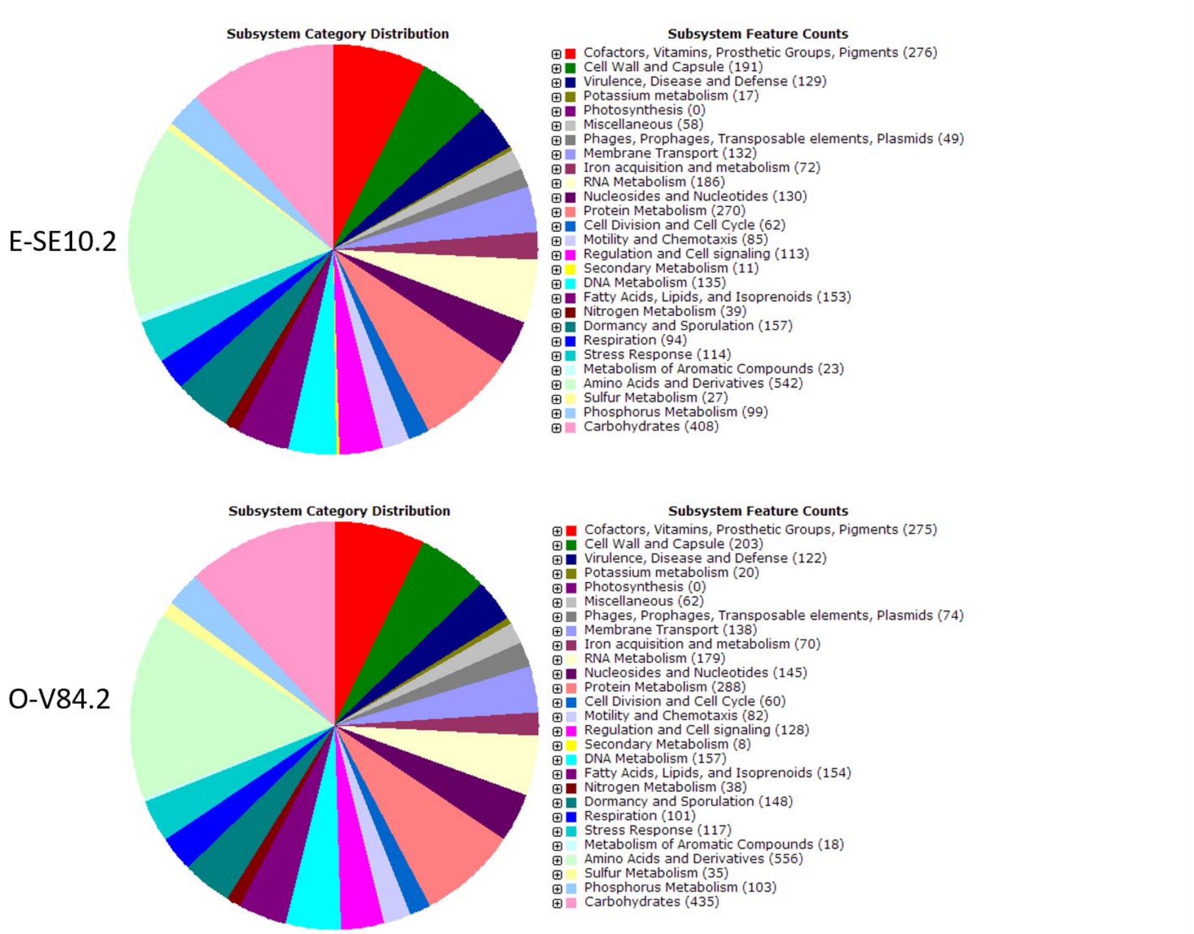

Figure S1. Subsystem category distribution of the *Bt* isolates E-SE10.2 and O-V84.2 by the genome annotation based on the Rast server. The right pie chart indicates the percentage or predicted encoding genes associated to at least one subsystem.

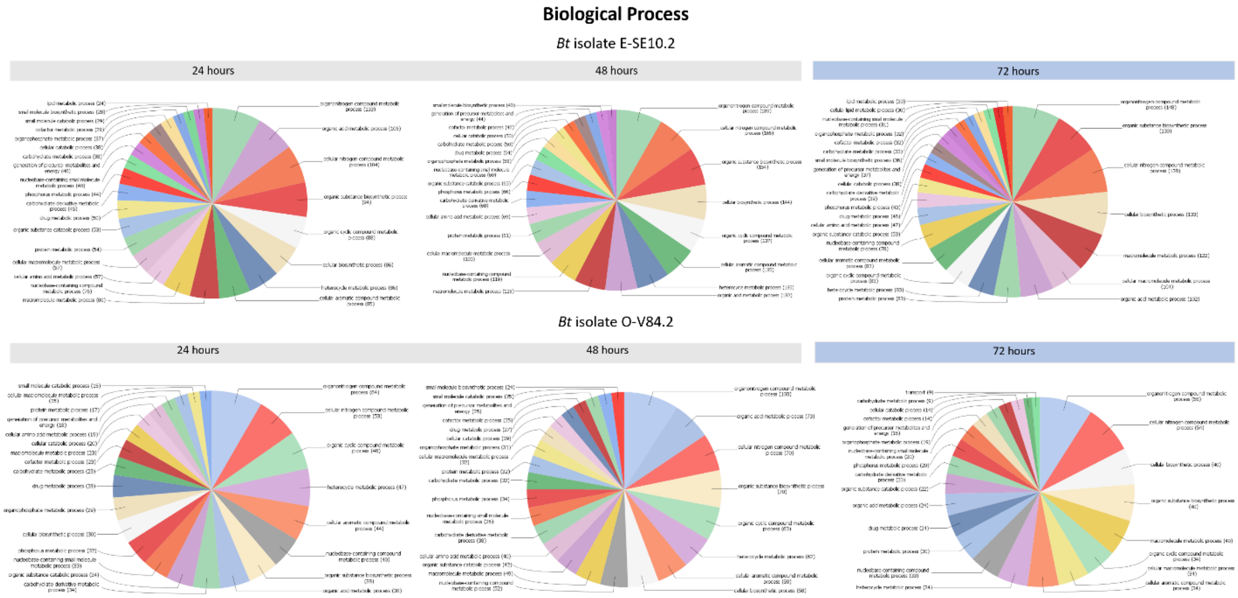

Figure S2. Functional annotation of the protein identification from the concentrated supernatants at 24 h and 48 h in LB medium, and of the solubilized proteins from the crystal at 72 h in CCY medium. The number of sequences that belong to the group are indicated in brackets.

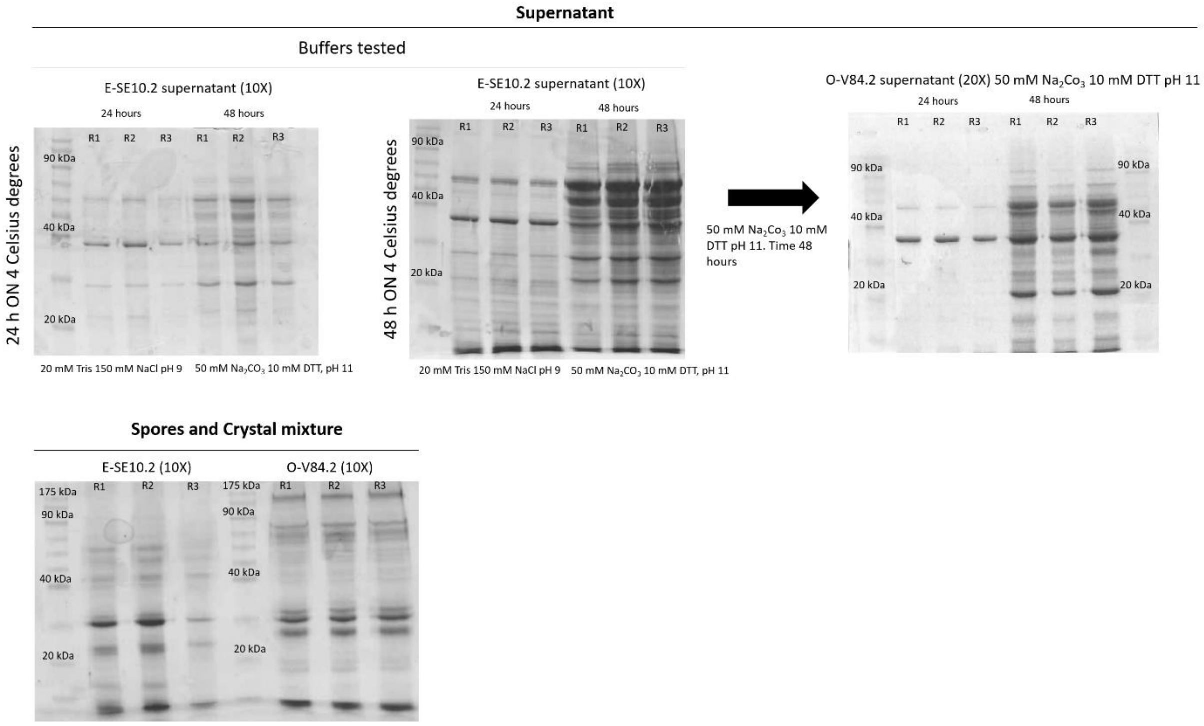

Figure S3. SDS-PAGE of the three replicates of the concentrated supernatants and solubilized proteins from the spore/crystal mixtures and growth curve of the *Bt* isolates E-SE10.2 and O-V84.2.
